# Features of Postpartum Hemorrhage-Associated Thrombotic Microangiopathy and Role of Short-Term Complement Inhibition

**DOI:** 10.1016/j.ekir.2024.01.035

**Published:** 2024-01-23

**Authors:** Jessica K. Kaufeld, Lucas Kühne, Ulf Schönermarck, Jan Hinrich Bräsen, Constantin von Kaisenberg, Bodo B. Beck, Florian Erger, Carsten Bergmann, A.N.K.E. von Bergwelt-Baildon, Paul T. Brinkkötter, Linus A. Völker, Jan Menne

**Affiliations:** 1 Department of Nephrology and Hypertension, Medical School Hannover, Hannover, Germany; 2 Department II of Internal Medicine and Center for Molecular Medicine Cologne (CMMC), Faculty of Medicine and University Hospital Cologne, University of Cologne, Cologne, Germany; 3 Cologne Cluster of Excellence on Cellular Stress Responses in Ageing-Associated Diseases (CECAD), Cologne, Germany; 4 Department of Medicine IV, Division of Nephrology, LMU University Hospital, LMU Munich, Munich, Germany; 5 Nephropathology Unit, Department of Pathology, Hannover Medical School, Hannover, Germany; 6 Department of Gynecology, Medical School Hannover, Hannover, Germany; 7 KRH Klinikum Mitte—Location Siloah, Hannover, Germany; 8 Institute of Human Genetics, Faculty of Medicine and University Hospital Cologne, University of Cologne, Cologne, Germany; 9 Center for Molecular Medicine Cologne (CMMC), University of Cologne, Faculty of Medicine and University Hospital Cologne, Cologne, Germany; 10 Medizinische Genetik Mainz, Limbach Genetics, Mainz, Germany

**Keywords:** complement inhibition, genetic kidney disease, p-aHUS, postpartum hemorrhage, pregnancy, renal cortical necrosis

## Abstract

**Introduction:**

In pregnancy-related atypical hemolytic uremic syndrome (p-aHUS), transferring recommendations for treatment decisions from nonpregnant cohorts with thrombotic microangiopathy (TMA) is difficult. Although potential causes of p-aHUS may be unrelated to inherent complement defects, peripartal complications such as postpartum hemorrhage (PPH) or (pre)eclampsia or Hemolysis, Elevated Liver enzymes and Low Platelets (HELLP) syndrome may be unrecognized drivers of complement activation.

**Methods:**

To evaluate diagnostic and therapeutic decisions in the practical real-life setting, we conducted an analysis of a cohort of 40 patients from 3 German academic hospitals with a diagnosis of p-aHUS, stratified by the presence (*n* = 25) or absence (*n* = 15) of PPH.

**Results:**

Histological signs of TMA were observed in 84.2% of all patients (100% vs. 72.7% in patients without or with PPH, respectively). Patients without PPH had a higher likelihood (20% vs. 0%) of pathogenic genetic abnormalities in the complement system although notably less than in other published cohorts. Four of 5 patients with observed renal cortical necrosis (RCN) after PPH received complement inhibition and experienced partially recovered kidney function. Patients on complement inhibition with or without PPH had an increased need for kidney replacement therapy (KRT) and plasma exchange (PEX). Because renal recovery was comparable among all patients treated with complement inhibition, a potential beneficial effect in this group of pregnancy-associated TMAs and p-aHUS is presumed.

**Conclusion:**

Based on our findings, we suggest a pragmatic approach toward limited and short-term anticomplement therapy for patients with a clinical diagnosis of p-aHUS, which should be stopped once causes of TMA other than genetic complement abnormalities emerge.

## Graphical abstract

**Figure:**
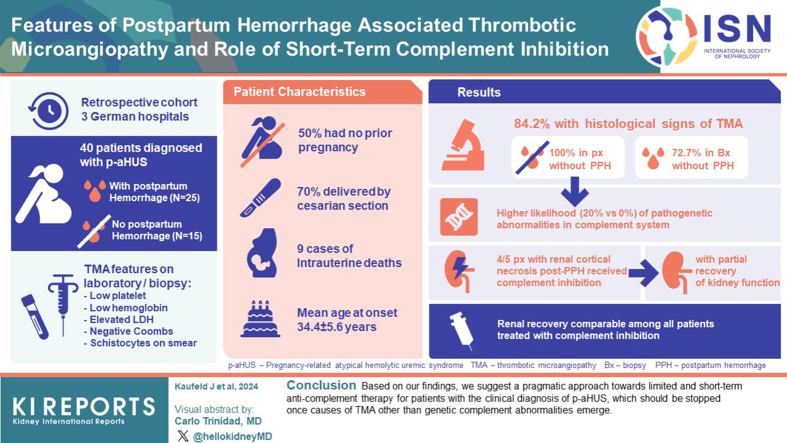

Pregnancy-related microangiopathic disorders encompass a spectrum of conditions ranging from (pre)eclampsia and HELLP syndrome to rare forms of TMAs such as thrombotic thrombocytopenic purpura (TTP) and p-aHUS.^1^, ^2^, ^3^ All TMA forms feature microangiopathic hemolytic anemia, thrombocytopenia in the presence of schistocytes, and ischemic end-organ damage. Preeclampsia and HELLP affect up to 8% of all pregnancies, whereas TTP and p-aHUS are rare with an estimated incidence of 1 in 25,000 pregnancies.^1^, ^2^, ^3^ Diagnosis and classification of TMA is challenging due to significant overlap in the clinical presentation as well as lack of specific diagnostic tests. The underlying etiology is often unknown at initial presentation.

National cohorts have identified susceptibility variants in complement genes in 41% to 86% of all patients with p-aHUS with poor renal outcomes.^4^, ^5^, ^6^ p-aHUS is now believed to be a continuum with atypical hemolytic uremic syndrome (aHUS) based on dysregulation, either acquired or inherited, of the complement system.^7^ In contrast, in (pre)eclampsia and HELLP syndrome, the underlying pathophysiology is thought to be a deficient placentation due to angiogenic imbalance and resulting in hypoperfusion of the fetoplacental unit. Delivery remains the only definitive management, and laboratory parameters should normalize within 48 to 72 hours. Anticomplement therapy is only recommended in pregnancy when the diagnosis of aHUS is certain^8^; however, the challenging task for the treating physician is to secure the diagnosis of p-aHUS.

Many obstetric complications such as (pre)eclampsia/HELLP, PPH, and cesarean delivery can be considered complement-activating conditions.^6^^,^^9^, ^10^, ^11^, ^12^ The delivery of the placenta during the postpartum phase eliminates its complement-inhibiting effect via CD55 (decay accelerating factor) and further predisposes for TMA.^4^^,^^13^^,^^14^ The heme molecule itself has been identified as complement activator in aHUS.^15^

Of these complications, PPH occurs frequently in about 6% of all deliveries.^16^ PPH is defined as a blood loss of more than 500 ml by the World Health Organization, but some authors contend that this may occur in up to 50% of all deliveries.^16^^,^^17^ In rare cases, PPH may not be straightforwardly recognized as a cause of TMA because it may also cause disseminated intravascular coagulation leading to thrombocytopenia. This conundrum is reflected in a recently published complex diagnostic algorithm of the international working group on pregnancy-related TMAs.^7^ Given that PPH may cause RCN with a low probability for renal recovery, this working group recommends excluding patients with PPH from further TMA diagnostic workup and focusing on screening for RCN without initiating immediate PEX or anticomplement therapy.^7^^,^^18^ In contrast, data from European cohorts suggest that missing the diagnosis of p-aHUS may exclude 41% to 86% of patients with peripartal TMA from potential organ-saving complement inhibitor therapy.^4^, ^5^, ^6^^,^^19^

To provide a basis for diagnostic and therapeutic decisions in the context of the complexities of pregnancy-related acute TMA, we conducted an analysis on a cohort of 40 patients of 3 German academic hospitals with the presumptive diagnosis of p-aHUS, stratified by the presence or absence of PPH. The data presented here complement the scarce available data on TMA in pregnancy and provide a basis for clinical decision-making despite the current and anticipated future lack of randomized trials in pregnancy-related TMAs.

## Methods

The patients were retrospectively identified at 3 German academic hospitals between 2015 and 2022. The inclusion criteria were laboratory results compatible with Coombs-negative microangiopathic hemolytic anemia during pregnancy or up to 2 weeks after delivery, with the main working diagnosis of p-aHUS. This excluded patients with known TTP, uncontrolled infections, particularly Shiga-toxin–producing pathogens, active malignancies, and known inborn or inherited defects in cobalamin or vitamin B12 metabolism. TMA was diagnosed based on the presence of a reduced platelet count (<100 × 10^9^/l), reduced hemoglobin level (<10 g/dl), elevated lactate dehydrogenase (>1.5 upper limit of normal), undetectable serum haptoglobin, negative direct erythrocyte antiglobulin (Coombs) test, presence of schistocytes on blood smear, and/or TMA features in a kidney biopsy. Acquired or congenital TTP could be ruled out in all patients by ADAMTS13 activity well above the reported cut-off of 10%.^20^ The patients were then stratified according to the presence of relevant PPH as judged by the treating physician.

Genetic testing was performed at the individual hospitals as standard-of-care according to local practice with either Sanger-sequencing or next-generation sequencing. Known pathogenic mutations hereafter referred to as susceptibility variants for aHUS as well as the presence of risk-related haplotypes (e.g., MCP-H2 and CFH-H3) were reported after the analysis of the following genes: *C3*, *CD46* (*MCP*), *CFB*, *CFH*, *CFHR1*, *CFHR3*, *CFHR4*, *CFI*, *DGKE*, and *THBD.* In 10 cases, one of the genetic departments did not report risk-related haplotypes in the absence of susceptibility variants for aHUS.

Descriptive statistical tests were performed using GraphPad Prism version 8.0.0 for Windows (GraphPad Software, San Diego, CA; www.graphpad.com). *P*-values were calculated using 2-sided unpaired *t* tests for quantitative variables with normal distribution, Mann-Whitney *U* test for quantitative variables without normal distribution, and Fisher exact test for categorical variables. This study was approved by local ethics committees of the involved academic hospitals.

## Results

### Demographics and Pregnancy-Associated Complications

Baseline characteristics as well as obstetric complications of the patients are summarized in Table 1. Individual patient data are presented in Supplementary Table S1. A total of 40 patients with pregnancy-associated TMA were included. Half of the patients had no previous pregnancy. Most patients delivered by cesarean delivery (70.0 %); among those, 53.5% were emergency procedures. Intrauterine deaths occurred in 9 cases, including 4 missed abortions before the 20th week of gestation. The length of follow-up varied widely and averaged 10.5 months overall.

**Table 1:** Baseline demographic data and peripartal complications; differentiation of patient characteristics in patients with or without PPH

Pregnancy-associated parameter	Total	PPH	no PPH	*P*-value
Baseline before delivery				
Number of patients, *n* (%)	40 (100.0)	25 (62.5)	15 (37.5)	
Age at onset (yr), mean ± SD	34.4 ± 5.6	35.6 ± 5.4	32.4 ± 5.5	0.08
Week of pregnancy, median (IQR)	36.0 (32.0–38.0)^a^	37.0 (35.0–40.0)^b^	32.5 (22.8–36.3)^c^	0.004
Previous pregnancies, median (IQR)	0.0 (0.0-2.0)	0.0 (0.0-2.0)	0.0 (0.0-2.0)	0.74
Zero, *n* (%)	22 (55.0)	13 (52.0)	9 (60.0)	0.62
≥1, *n* (%)	15 (37.5)	9 (36.0)	6 (40.0)	0.80
no data, *n* (%)	3 (7.5)	3 (12.0)	0 (0.0)	0.28
(pre)eclampsia/HELLP, *n* (%)	12 (30.0)	7 (28.0)	5 (33.3)	0.74
Medical history				
aHUS, *n* (%)	2 (5.0)	0 (0.0)	2 (13.3)	0.14
autoimmune disease, *n* (%)	6 (15.0)	3 (12.0)	3 (20.0)	0.65
Malignancy, *n* (%)	1 (2.5)	0 (0.0)	1 (6.7)	0.38
Delivery				
Vaginal delivery, *n* (%)	12 (30.0)	7 (28.0)	5 (33.3)	0.74
Cesarean delivery (CD), *n* (%)	28 (70.0)	18 (72.0)	10 (66.7)	0.74
As emergency procedure, *n* (% of CD)	15/28 (53.5)	8/18 (44.4)	7/10 (70.0)	0.25
Fetal complication				
Intrauterine fetal death/abortion	9 (22.5)	6 (24.0)	3 (20.0)	1.00

CD, cesarean delivery; HELLP, Hemolysis, Elevated Liver enzymes and Low Platelets syndrome; IQR, interquartile range; PPH, postpartum hemorrhage

a Four cases before week 20, 7 data points missing.

b One case before week 20, 5 data points missing.

c Three cases before week 20, 2 data points missing.

Major bleeding complications with PPH preceded TMA in 25 patients (62.5%) (PPH group), whereas TMA occurred in 15 patients without PPH (no PPH group). PPH was related to uterine atony in combination with preceding preeclampsia (*n* = 7), intrauterine fetal death (*n* = 6), missed abortion (*n* = 2), and cesarean delivery (*n* = 18). At least one of these known risk factors for PPH was identified in 19 (76.0%) women with PPH.

The mean age was 35 and 32 years in the PPH and no PPH groups, respectively, slightly above the average age for pregnancy in Germany.^21^ Patients without PPH presented earlier than those with PPH (33rd and 37th week of gestation, *P* = 0.004).

### TMA

**Figure 1:**
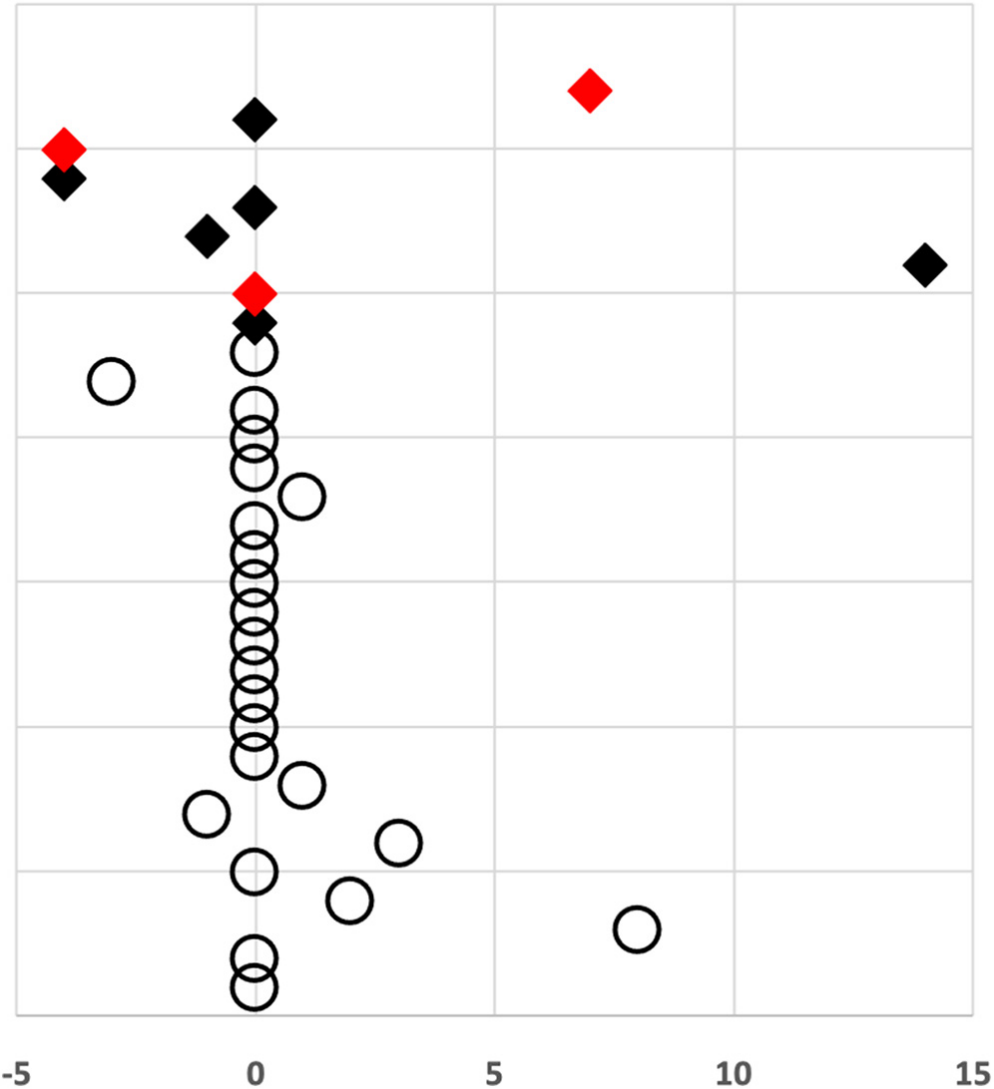
Temporal correlation of the thrombotic microangiopathic (TMA) event before and after delivery (day 0). Red symbols indicate individuals, in whom a pathogenic mutation of a complement regulatory gene could be identified. Patients without postpartum hemorrhage are represented by a solid rhombus. Patients with postpartum hemorrhage are depicted in circles. Most TMAs occurred at the time of delivery or shortly thereafter. In 7 patients, the exact onset of event could not be determined especially when (pre)eclampsia was documented and in 1 patient onset was 10 weeks before delivery.

Most TMA episodes started immediately at time of delivery or within 2 weeks into the postpartum period (65.0%) (Figure 1). Only 1 patient with a known underlying IgA-nephritis presented 10 weeks before delivery (case 7, Supplementary Table S1). At onset, the average lactate dehydrogenase level across all patients was 2037 ± 1309.2 U/l. Platelets reached an average nadir of 46.9 ± 32.3 × 10E^9^/l, with an average hemoglobin concentration of 6.0 ± 1.4 g/dl. The TMA-related laboratory parameters in patients with PPH did not differ significantly from those in patients without PPH (Table 2).

**Table 2:** Clinical parameters of aHUS episode and postpartal clinical course; differentiation of patient characteristics in patients with or without PPH

TMA-associated parameter	Total	PPH	no PPH	*P*-value
Number of patients, *n* (%)	40 (100)	25 (62.5)	15 (37.5)	
TMA signs and symptoms				
Min. platelet (× 10^3^/μl), mean ± SD	46.9 ± 32.3	45.9 ± 29.8	48.8 ± 37.8	0.80
Min. Hb (g/dl), mean ± SD	6.0 ± 1.4	5.7 ± 1.0	6.6 ± 2.0	0.06
Max. LDH (U/l), mean ± SD	2037.3 ± 1309.2	2100.1 ± 1275.0	1906.33 ± 1426.5	0.68
Max. creatinine mg/dl, median (IQR)	5.2 (4.0–10.3)	5.3 (4.2–9.9)	5.2 (1.9–11.9)	0.72
Neurological involvement, *n* (%)	8 (20.0)	5 (20.0)	3 (20.0)	1.00
Treatment modalities				
Need for acute KRT, *n*/*N* (%)	24/40 (60.0)	16/20 (64.0)	8/15 (53.3)	0.51
Duration of KRT (wk), median (IQR)	3.9 (1.3–20.0)	3.0 (0.9–9.9)	7.6 (1.6–25.0)	0.44
KRT stop within 30 ds, *n*/*N* (%)	12/24 (50.0)	9/16 (56.2)	3/8 (37.5)	0.66
Receiving PEX treatment, *n*/*N* (%)	30/40 (75.0)	20/25 (80.0)	10/15 (66.7)	0.46
# of PEX, median (IQR)	4.0 (3.0–6.0)	4.0 (2.0–5.0)	5.0 (3.0–7.5)	0.25
Complement inhibitor treatment (CIT)				
CIT treatment, *n*/*N* (%)	29/40 (72.5)	19/25 (76.0)	10/15 (66.7)	0.72
CIT start after event (d), median (IQR)	10.0 (6.0–16.0)	10.0 (7.0–14.0)	12.0 (4.0–29.5)	0.54
Duration of CIT treatment (wk), median (IQR)	21.8 (11.5–42.6)	17.9 (11.6–51.2)	25.7 (0.9–43.7)	0.85
CIT ongoing at end of FU, *n*/*N* (%)	5/29 (17.2)	2/19 (10.5)	3/10 (30.0)	0.30
Biopsy				
Kidney biopsy *n*/*N* (%)	19/40 (47.5)	11/25 (44.0)	8/15 (53.3)	0.57
TMA in biopsy, *n*/*N* (%)	16/19 (84.2)	8/11 (72.7)	8/8 (100.0)	0.23
Renal cortical necrosis (RCN)				
Patchy RCN, *n*/*N* (%)	2/19 (10.5)	2/11 (18.1)	0/8 (0.0)	0.48
Diffuse RCN, *n*/*N* (%)	3/19 (15.7)	3/11 (27.2)	0/8 (0.0)	0.22
Genetics				
Genetic testing, *n*/*N* (%)	37/40 (92.5)	25/25 (100.0)	12/15 (80.0)	0.046
Susceptibility (pathogenic) variant, *n*/*N* (%)	3/37 (8.1)	0/25 (0.0)	3/12 (25.0)	0.028
Homozygous *CFHR1* und *CFHR3* deletion, *n*/*N* (%)	2/37 (5.4)	1/25 (4.0)	1/12 (8.3)	1.00
Patients with at least 1 risk-related haplotype when reported, *n*/*N* (%)^a^	15/27 (55.5)	9/17 (52.9)	6/10 (60.0)	1.00
Follow-up				
Time of FU in months, median (IQR)	10.5 (2.0–19.0)	15.0 (2.0–20.5)	7.0 (2.0–16.0)	0.49
eGFR at end of FU (ml/min per 1.73 m^2^; CKD-EPI), mean ± SD	61.4 ± 36.4	63.3 ± 35.6	58.3 ± 38.8	0.68
CKD stage at end of follow-up				
eGFR >60 ml/min per 1.73 m^2^, *n*/*N* (%)	25/40 (62.5)	16/25 (64.0)	9/15 (60.0)	0.80
eGFR 30–59 ml/min per 1.73 m^2^, *n*/*N* (%)	6/40 (15.0)	3/25 (12.0)	3/15 (20.0)	0.65
eGFR 15–29 ml/min per 1.73 m^2^, *n*/*N* (%)	4/40 (10.0)	4/25 (16.0)	0/15 (0.0)	0.28
ESKD at end of FU, *n*/*N* (%)	5/40 (12.5)	2/25 (8.0)	3/15 (20.0)	0.51
Patients with relapse after cessation of treatment during course of FU, *n*/*N* (%)	1/40 (2.5)	0/25 (0.0)	1/15 (6.7)	0.38

CFH, complement factor H; CIT, complement inhibitor treatment; CKD, chronic kidney disease; CKD-EPI, chronic kidney disease-epidemiology collaboration; eGFR, estimated glomerular filtration rate; ESKD, end-stage kidney disease; FU, follow-up; IQR, interquartile range; KRT, kidney replacement therapy; LDH, lactate dehydrogenase; Max., maximum; Min., minimum; PEX, plasma exchange; PPH, postpartum hemorrhage; RCN, renal cortical necrosis; TMA, thrombotic microangiopathy.

a In 10 patients (8 in the PPH and 2 in the no-PPH group) there was no reporting of risk haplotypes.

There was significant renal impairment in all patients, with a maximum median creatinine of 5.2 (4.0–10.3) mg/dl. Neurological abnormalities were reported in 20% of all cases, ranging from mild symptoms of agitation or confusion (*n* = 2) and visual disturbances (*n* = 1) to more severe symptoms such as seizures (*n* = 4) and stroke (*n* = 1).

### Kidney Biopsies

Kidney biopsies were performed in 19 of 40 cases (47.5%). Contraindications such as a very low platelet count or missing patient consent were reported in most cases without a kidney biopsy. Notably, there was no difference in the frequency of kidney biopsies between the PPH and non-PPH groups.

Histological signs of acute, subacute, or chronic TMA were reported in 16 patients (84.2 %); in 100% of all biopsies in the no-PPH group versus 72.7% in the patient group with PPH (Table 3). Signs of underlying renal diseases other than TMA were detected in only 2 patients in the no-PPH group: IgA-nephritis in 1 patient with known IgA nephropathy (case 7, Supplementary Table S1) and 1 patient with systemic lupus erythematosus with a history of catastrophic antiphospholipid-syndrome (case 11, Supplementary Table S1). Five patients were histologically diagnosed with diffuse (3 cases) or patchy (2 cases) RCN. TMA could not be histologically confirmed in 3 of the 11 cases with PPH. These patients were diagnosed as aHUS by exclusion based on the presence of microangiopathic hemolytic anemia with acute renal failure.

**Table 3:** Characteristics of patients with postpartum hemorrhage (PPH) according to the use of CIT

Patient variables	Total	no CIT	with CIT	*P*-value
Number of patients with PPH, *n* (%)	25 (100.0)	6/25 (26.1)	19/25 (73.9)	
TMA signs and symptoms				
Min. platelet (× 10^3^/μl), mean ± SD	45.9 ± 29.8	45.2 ± 14.7	46.1 ± 33.9	0.95
Min. Hb (g/dl), mean ± SD	5.7 ± 1.0	5.9 ± 0.6	5.6 ± 1.1	0.61
Max. LDH (U/l), mean ± SD	2100.1 ± 1275.0	2109.0 ± 1293.9	2097.3 ± 1304.8	0.99
Max. creatinine mg/dl, median (IQR)	5.3 (4.2–9.9)	6.4 (4.6–81.8)	4.9 (4.0–10.3)	0.56
Treatment modalities				
Receiving PEX treatment, *n*/*N* (%)	20/25 (80.0)	3/6 (50.0)	17/19 (89.5)	0.07
Need for acute KRT, *n*/*N* (%)	16/25 (64.0)	2/6 (33.3)	14 (73.7)	0.14
Duration of KRT (wk), median (IQR)	3.0 (0.9–9.9)	0.7 (0.7–0.7)	3.9 (1.3–13.4)	0.17
Renal recovery after KRT, *n*/*N* (%)	14/16 (87.5)	1/2 (50.0)	13/14 (92.8)	0.24
eGFR at end of FU (ml/min per 1.73 m^2^; CKD-EPI), mean ± SD	63.3 ± 35.6	52.7 **±** 35.6	66.6 ± 35.9	0.41
CKD stage at end of follow-up				
eGFR >60 ml/min, *n*/*N* (%)	16/25 (64.0)	4/6 (66.7)	12/19 (63.2)	1.00
eGFR 30–59 ml/min, *n*/*N* (%)	3/25 (12.0)	0/6 (0.0)	3/19 (15.8)	0.55
eGFR 15–29 ml/min, *n*/*N* (%)	4/25 (16.0)	1/6 (16.7)	3/19 (15.8)	1.00
ESKD at end of FU, *n*/*N* (%)	2/25 (8.0)	1/6 (16.7)	1/19 (5.3)	0.10

CIT, complement inhibitor treatment; CKD, chronic kidney disease; eGFR, estimated glomerular filtration rate; ESKD, end-stage kidney disease; FU, follow-up; IQR, interquartile range; KRT, kidney replacement therapy; LDH, lactate dehydrogenase; Max., maximum; Min., minimum; PEX, plasma exchange; TMA, thrombotic microangiopathy.

### Renal Outcomes and KRT

KRT was required in 60.0% of all patients (64.0% of patients with PPH vs. 53.3% without PPH). KRT was continued for a median of 3.9 (1.3–20.0) weeks. Five patients developed end-stage kidney disease (ESKD) at the end of follow-up. In 1 patient, KRT was temporarily stopped but reinitiated when kidney function declined further. At the end of follow-up, 37.5% of all patients remained at chronic kidney disease stage 3 or worse, 62.5% at chronic kidney disease stage 2 or better with an average estimated glomerular filtration rate of 61.4 ml/min per 1.73 m^2^ overall as calculated using the chronic kidney disease-epidemiology collaboration formula.^22^ Owing to the infrequent occurrence of ESKD at the end of follow-up, we could not detect a statistical difference between the patient groups with and without PPH.

### Treatment With PEX and Complement Inhibitor Therapy

PEX was initiated in 75.0% of cases according to local practice. Patients treated with PEX received a median of 4 (3.0–6.0) treatments.

Complement inhibitor treatment (CIT with eculizumab; 1 patient in the PPH group received ravulizumab) was used in 72.5% (*n* = 29) of all patients (Table 2). Treatment was started after a median of 10 days after the first manifestation of TMA and was terminated after a median treatment duration of 21.8 weeks. Only 1 patient in the no-PPH group relapsed after the cessation of eculizumab treatment. This patient was later diagnosed with 2 aHUS susceptibility variants in the gene encoding for complement factor H in addition to a pathogenic thrombomodulin variant (case 8, Supplementary Table S1) and restarted on eculizumab for long-term therapy. Patients on complement inhibition were more likely to receive acute KRT (20/29, 68.9 %; *P* = 0.08 for difference between groups) or PEX (25/29, 86.2%; *P* = 0.014 for difference between both groups (Supplementary Table S2). Despite higher rates of dialysis dependence at presentation and longer time on KRT (4.1 weeks vs. 1.6 weeks) in the group on complement inhibitor therapy, renal outcome was comparable. Among patients receiving KRT (*n* = 24), 1 of 4 patients without complement inhibitor therapy (25.0 %) remained on KRT at the end of follow-up versus 4 of 20 patients treated on complement inhibitor therapy (20.0%) (Supplementary Table S2).

To understand the role of complement inhibitor therapy in PPH, we stratified all patients with PPH (*n* = 25) according to CIT. Nineteen of 25 patients with PPH received complement inhibitor therapy, whereas 6 patients were treated without complement inhibitor therapy. Hemoglobin, lactate dehydrogenase, and platelet count as indicators of TMA severity did not differ between the subgroups (Table 3). However, most patients on complement inhibitor therapy also received PEX compared with only half of the patients without it (17/19, 89.5% vs. 3/6, 50%; *P* = 0.07 for difference between groups) (Table 3). The outcome among patients with PPH was mostly favorable. In accordance with the results of the whole patient group, renal outcome was comparable despite higher rates of acute KRT at presentation and longer time on KRT (3.9 weeks vs. 0.7 weeks) in the CIT group (Table 3). In the group without PPH, similar rates for the need for PEX treatment as well as acute KRT were seen (Supplementary Table S3).

### Outcome of Patients With RCN

The diagnosis of RCN was based on histology in 5 of 19 patients with a kidney biopsy. All patients presented after an episode of PPH and needed acute KRT. PEX and complement inhibition were used in 3 patients, whereas PEX only and CIT only were used in 1 patient each. All 4 patients with RCN on kidney biopsy and after treatment with complement inhibition partially recovered kidney function, whereas only 1 patient (20.0%) without CIT developed ESKD (Supplementary Table S1 with description of individual clinical courses). KRT was discontinued after 5 months (patient 16), 9.5 months (patient 22), 2.5 weeks (patient 32), and 9 months (patient 39); and kidney function recovered to an estimated glomerular filtration rate of 20 to 33 ml/min per 1.73 m^2^.

### Outcome of Patients With Concurrent Diagnosis of Preeclampsia and HELLP

Twelve patients (30%) (*n* = 5 [33.3%] in the no PPH; and *n* = 7 [28%] in the PPH group) were admitted with the working diagnosis of preeclampsia or HELLP syndrome (Table 1.). Among the patients without PPH, 2 needed acute KRT and only 1 received complement inhibitor therapy and PEX without need for dialysis. All 5 patients recovered kidney function. Among the 7 patients with PPH and preeclampsia or HELLP, 5 received complement inhibitor therapy, of which 4 needed acute KRT with 1 remaining on KRT (Supplementary Table S1). Notably, 3 patients with preeclampsia or HELLP and PPH also experienced RCN after PPH, and 1 patient without complement inhibitor therapy remained dialysis-dependent at the end of follow-up (Supplementary Table S1).

### Complement Diagnostic and Genetic Workup

Individual complement components were measured in the acute setting in a small subset of a maximum of 23 patients. We observed an activation of the alternative complement pathway with isolated low C3 levels and increased concentrations of the terminal complement complex sC5b-9 in about 70% of the patients (Supplementary Table S4). There was no significant difference between patient subgroups (PPH vs. no PHH).

Genetic analysis of complement genes in most patients was performed according to local practice (*n* = 37, 92.5%). Susceptibility variants for aHUS in the aHUS-related complement genes were not found in any of the patients in the PPH group (0/25), whereas in 25.0% of patients in the no-PPH group (3/12), a pathogenic complement factor H mutation (CFH) was detected (*P* = 0.02 vs. PPH group). An additional thrombomodulin variant (c.127G>A) was observed in 1 patient in the latter group.

Two patients (1 in the PPH group and 1 in the no-PPH group) were diagnosed with homozygous *CFHR1* and *CFHR3* deletions. Without detectable anti-CFH autoantibodies, these patients were not counted toward the group carrying a susceptibility variant.^23^ Risk-related haplotypes (*MCP-H2* in 11 patients, *CFH-H3* in 3 patients, and *CFHR1∗B* in 2 patients; Supplementary Table S1) were identified alone or together with other haplotypes in a total of 15 of 37 (40.5%) patients (36.0% of PPH vs. 50.0% no PPH, *P* = 1.0 for difference between groups). This included the 3 patients with the additionally identified aHUS susceptibility variants as well as 1 patient with a variant of unknown significance of the C3-gene (case no 9, Supplementary Table S1). In 10 patients, no risk-related haplotypes were reported by the corresponding laboratory because no susceptibility variant was detected (Supplementary Table S1). Therefore, the overall incidence for risk-related haplotypes may be underestimated in this analysis.

## Discussion

Diagnosis and treatment of TMAs during pregnancy and in the postpartum period remain challenging, and the diagnosis is still primarily based on the clinical presentation. More common conditions such as (pre)eclampsia and HELLP syndrome must be separated from rare forms such as TTP and p-aHUS. Although a recent consensus statement from the international working group for pregnancy-related TMAs proposed a diagnostic workup and treatment recommendations for women with TMA related to pregnancy, these are inferred from conditions outside pregnancy, and evidence from randomized studies is lacking.^7^ Because patients are often admitted from other hospitals to the university clinics, crucial information about laboratory results before delivery and/or patient history as well as given therapy during the acute event before admission are often lacking. Moreover, clinicians are frequently confronted with TMA in the context of significant peripartal bleeding and require guidance on how to adjudicate these situations.

With our cohort study of 40 patients with pregnancy-associated aHUS, we aim to add information to this rare disease entity. All patients were diagnosed and treated according to routine clinical standard, with or without complement inhibitor therapy and a thorough diagnostic workup and follow-up, including a high rate of kidney biopsies and genetic testing in nearly all patients. The histopathologic diagnosis of TMA in 84.2% of all patients with a kidney biopsy aligns well with the clinical diagnosis of p-aHUS. Despite severe kidney involvement and need for acute KRT in 60% of all patients, renal and patient outcomes were favorable in this cohort. Five patients (12.5%), however, remained in ESKD at the end of follow-up. Notably, none of the patients who were dialysis-dependent at follow-up had any inherent susceptibility aHUS variants (with genetic workup not done in 1 patient).

Patients were stratified according to the presence of PPH (PPH vs. no-PPH group), a known potent trigger of complement activation (so-called complement-amplifying condition). Clinical TMA severity, as well as the use of PEX, CIT, or need for acute KRT were similar in both groups. Patients with PPH treated with complement inhibition seemed more likely to stop dialysis, despite higher rates of acute dialysis at presentation and longer time on dialysis indicating more severe disease presentation.

RCN was diagnosed in 5 patients with PPH. All patients initially required acute KRT. Kidney function recovered in 4 patients (80%) treated with CIT, and 1 patient developed ESKD without use of CIT. In a historic cohort of 64 obstetric patients in India with RCN diagnosed by histopathologic examination, 19.5% were due to postabortal sepsis, 13.3% to abruptio placentae, 10.5% to PPH, 6.2% to puerperal sepsis, and 7.1% to eclampsia, demonstrating the large overlap of complement-activating conditions in pregnancy.^24^ Notably, histopathologic findings of diffuse or patchy ischemic lesions in RCN share some similarities with the histological signs of aHUS, and it should be remembered that in the initial description of hemolytic uremic syndrome by Gasser, all 5 affected children had bilateral RCN.^25^ Moreover, acute blood loss as seen in patients with PPH activates both the complement and coagulation cascade, and laboratory features of TMA and disseminated intravascular coagulation do significantly overlap.^26^ Disseminated intravascular coagulation, therefore, remains an important differential in patients with PPH. In the acute situation, incomplete or pending laboratory results and diagnostic tests from the initial presentation may prevent the definite safe exclusion of disseminated intravascular coagulation as a cause of RCN and TMA after PPH. In our cohort, all 5 patients with RCN also had finding of TMA in the biopsy and were treated as p-aHUS.

A recent report on 18 cases of RCN related to PPH with acute kidney injury and initial need for KRT demonstrated that 8 of 18 patients reached ESKD, whereas KRT could be stopped in 10 patients, more than 50% of all patients with RCN, although none recovered normal renal function.^18^ Laboratory signs of TMA (thrombocytopenia and hemolysis) remitted within 2 to 13 days, indicating a prolonged time of complement-activation that may be shortened by complement inhibitor therapy. The recent consensus statement from the international working group for pregnancy-related TMA^7^ proposed for patients with significant peripartal bleeding using magnetic resonance imaging or ultrasound to rule out RCN. If RCN is present, it is suggested to proceed conservatively under the assumption of a dismal renal prognosis with 30% to 50% of all patients progressing to ESKD.^27^^,^^28^ The data from our cohort suggest that anticomplement therapy may improve kidney survival if used in the initial phase of strong complement activation immediately after the inciting event. Certainly, interventional, prospective trials are needed to test this hypothesis.

Several cohorts involving 21 to 87 patients with the diagnosis of p-aHUS report genetic abnormalities in 41% to 86% with excellent response to eculizumab or poor renal prognosis in the pre-eculizumab era.^4^, ^5^, ^6^^,^^19^ The low yield of genetic susceptibility variants for aHUS (25% without and 0% with PPH) in our cohort reflects differences in the clinical setting and different inclusion criteria. Other published cohorts are heavily preselected and exclude patients with concomitant PPH or autoimmune disorders. Our cohort more closely reflects real-world scenarios a consulting physician may encounter. Genetic susceptibility variants for aHUS were primarily found in patients without PPH (25%) and at the CFH locus, but none in the PPH group.

In primary aHUS as the main form of complement-mediated TMA, dysregulation of the alternative pathway is the driving force of endothelial damage and has been associated with genetic susceptibility factors related to the complement system. Moreover, aHUS likely results from interactions between genetic susceptibility factors in the complement system and environmental factors (e.g., infections, pregnancy, or injuries) that trigger complement activation and/or endothelial cell damage (so-called complement-amplifying conditions). Stronger genetic risk factors confer a lower threshold for external factors to trigger TMA and vice versa. With PPH as a strong complement-activating trigger, less genetic influence may be necessary. In addition, intrauterine complement activation via C5a in response to amniotic fluid or any fetal material has been described as an important trigger of postpartum acute myometritis and uterine atony.^29^ This suggests that patients with a strongly activated complement system may be predisposed to PPH. Of note, 2 patients in the no-PPH group were diagnosed with complement-mediated TMA related to underlying disease (IgA and systemic lupus erythematosus) but were counted toward the p-aHUS cohort and treated with CIT (Supplementary Table S1). The decision to initiate CIT was based on the clinical course and severeness of the clinical presentation.

**Figure 2:**
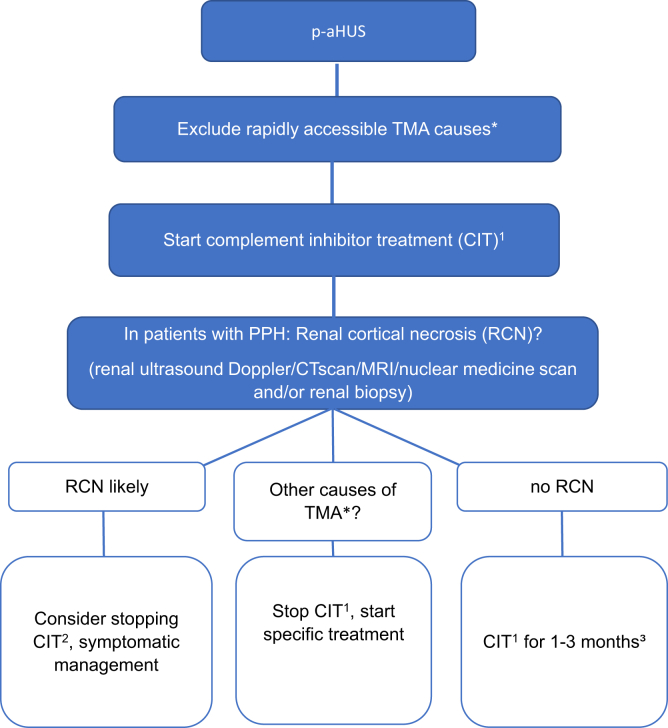
Pragmatic treatment algorithm for patients with p-aHUS and postpartum hemorrhage. ^1^In countries with no access to CIT, an initial treatment trial with plasma exchange should be considered in women requiring hemodialysis. All patients should be planned for genetic testing to search for aHUS susceptibility variants. ^2^Considering the extent of RCN, kidney function parameters and histopathologic findings, CIT should be continued for 1 to 3 months as per clinical judgment. ^3^In patients with a detectable susceptibility variant for aHUS, treatment may be continued depending on kidney function and individual risk for relapse (genetics, trigger factors). ∗Other causes of TMA may emerge after turn-around of diagnostic tests (e.g., aTTP, autoimmune disorders, infections). CIT, complement inhibitor treatment; p-aHUS, pregnancy-induced atypical hemolytic syndrome; PPH, postpartum hemorrhage; RCN, renal cortical necrosis; TMA, thrombotic microangiopathy; TTP, acquired thrombotic thrombocytopenic purpura.

Timely therapeutic complement inhibition is warranted in complement-mediated TMA forms to increase the chances of kidney survival. However, the prevalence of complement-mediated TMA in real-world scenarios is low. Therefore, it seems plausible that the duration of complement inhibitor therapy should be tailored independent of the occurrence of PPH. We suggest that complement therapy should be stopped in the following scenarios given that no susceptibility variants were identified on genetic testing: (i) with the detection of other causes of TMA in need for different therapeutic strategies, (ii) within 3 months when no improvement of kidney function occurs, and (iii) ideally with complete recovery of kidney function or stabilization of kidney function. The duration of treatment is unclear in patients with the finding of RCN on kidney biopsy. As a general recommendation, treatment may be stopped if there is no improvement of kidney function during the first 3 months. In patients with a detectable genetic susceptibility variant for aHUS, treatment should be prolonged at least until estimated glomerular filtration rate and proteinuria have stabilized for 3 months and then guided by the individual disposition for relapse (Figure 2).

Considering the risks, therapy with complement inhibition has been associated with severe bacterial infections, particularly by capsulated pathogens; however, mitigation strategies and experience with complement inhibitor therapy have decreased the risk of these complications. Although eculizumab has not been approved for use in pregnancy, it has been safely used in this setting with positive renal and pregnancy outcomes for both fetus and mother.^4^

The small sample size, low statistical power, and retrospective nature without a uniform diagnostic or therapeutic protocol are limitations of this study. The cohort reflects only patients identified at 3 academic hospitals in Germany, which limits its ability to extrapolate the findings to other settings and countries, particularly countries with significantly higher birth rates and different demographics. Reimbursement of costly complement inhibitor therapies may prevent adoption of our proposed approach in low-resource settings.

### Conclusion

Caring for patients with pregnancy-related TMA is complex. A variety of differential diagnoses need to be considered, and individualized treatments may range from best supportive care to therapeutic PEX and/or use of complement inhibitor therapy. A high likelihood of susceptibility variants for aHUS in complement genes from patients diagnosed with p-aHUS has been suggested.^5^ In addition, current recommendations systematically exclude pregnant patients with TMA following a peripartal bleeding complication from considerations for CIT. However, during pregnancy, patients may accumulate multiple complement-activating factors, and there may be a strong rationale for the use of complement inhibitors such as eculizumab for all patients with p-aHUS, even when bleeding is present and testing for genetic abnormalities often returns negative results. Duration of CIT should be individualized according to the extent of RCN, the dynamics of renal recovery, and outcome of further diagnostics. Collecting data on pregnancy-related TMAs such as p-aHUS is essential to improve our understanding of this rare entity, and prospective trials are warranted to investigate therapeutic complement inhibition in this vulnerable patient group.

## Disclosure

BBB received consulting fees from Alnylam pharmaceuticals. ABB received grants from Alexion/AstraZeneca, Chemocentry/Vifor, Ablynx/Sanofi, and Allena Pharmaceuticals. CB has received consulting fees from Alexion and PTC and grants from DFG, BMBF, and Limbach Group. LAV reports grants from the Else-Kroener-Fresenius Stiftung (2015_A224) and research funding, travel support, and consulting fees from Alexion, AstraZeneca, Bayer, GC Biopharm, and Sanofi-Genzyme. JM received personal fees from Alexion, SanofiGenzyme, and Ablynx (speaker honoraria, advisory boards). JKK reports speaker honoraria and advisory boards from Alexion, Sanofi, Takeda, Amicus, Chiesi, and Novartis. PTB reports grants from the German Research Foundation (BR2955/8) during the conduct of the study and personal fees from Alexion, Astellas, Bayer, Sanofi Genzyme, Pfizer, and Vifor (speaker honoraria, advisory boards). US has received grants from Alexion/AstraZeneca, Chemocentry/Vifor, Ablynx/Sanofi, Allena Pharmaceuticals and consulting fees from Chemocentryx/Vifor, Travere/Vifor, Ablynx/Sanofi, and Alnylam Pharmaceuticals. All the other authors declared no competing interests.

## Supplementary Material

Supplementary File (PDF)

**Table S1.** Individual patient characteristics including Individual courses and outcomes of patients presenting RCN on biopsy.

**Table S2.** Patient characteristics in women with and without complement inhibitor treatment.

**Table S3.** Patient characteristics in women in the no PPH group, with and without complement inhibitor treatment.

**Table S4.** Complement laboratory in a subset of patients.
