# Erratum to “Features of Postpartum Hemorrhage-Associated Thrombotic Microangiopathy and Role of Short-Term Complement Inhibition” [*Kidney International Reports* Volume 9, Issue 4, April 2024, Pages 919-928]

**DOI:** 10.1016/j.ekir.2024.08.001

**Published:** 2024-08-06

**Authors:** Jessica K. Kaufeld, Lucas Kühne, Ulf Schönermarck, Jan Hinrich Bräsen, Constantin von Kaisenberg, Bodo B. Beck, Florian Erger, Carsten Bergmann, Anke von Bergwelt-Baildon, Paul T. Brinkkötter, Linus A. Völker, Jan Menne

**Affiliations:** 1 Department of Nephrology and Hypertension, Medical School Hannover, Hannover, Germany; 2 Department II of Internal Medicine and Center for Molecular Medicine Cologne (CMMC), Faculty of Medicine and University Hospital Cologne, University of Cologne, Cologne, Germany; 3 Cologne Cluster of Excellence on Cellular Stress Responses in Ageing-Associated Diseases (CECAD), Cologne, Germany; 4 Department of Medicine IV, Division of Nephrology, LMU University Hospital, LMU Munich, Munich, Germany; 5 Nephropathology Unit, Department of Pathology, Hannover Medical School, Hannover, Germany; 6 Department of Gynecology, Medical School Hannover, Hannover, Germany; 7 KRH Klinikum Mitte—Location Siloah, Hannover, Germany; 8 Institute of Human Genetics, Faculty of Medicine and University Hospital Cologne, University of Cologne, Cologne, Germany; 9 Center for Molecular Medicine Cologne (CMMC), University of Cologne, Faculty of Medicine and University Hospital Cologne, Cologne, Germany; 10 Medizinische Genetik Mainz, Limbach Genetics, Mainz, Germany

The authors regret that the author’s name Anke von Bergwelt-Baildon was incorrectly published as A.N.K.E. von Bergwelt-Baildon, and there was an error in the graphical abstract abbreviating biopsy as px instead of Bx. The corrected graphical abstract is shown below.

**Figure:**
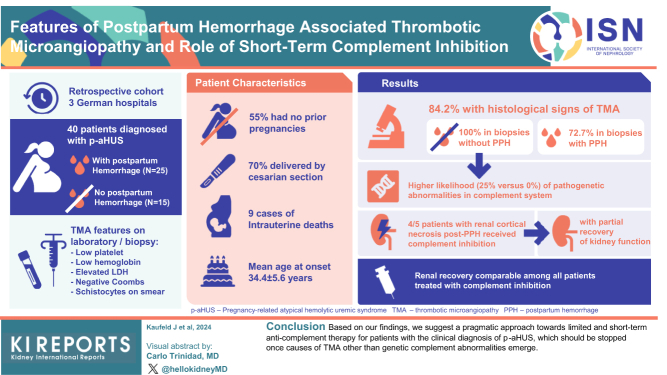

We apologize for any inconvenience caused.

DOI of original article: https://doi.org/10.1016/j.ekir.2024.01.035

