# Supplementary material for: Features of Postpartum Hemorrhage-Associated Thrombotic Microangiopathy and Role of Short-Term Complement Inhibition
Source: Kidney Int Rep. 2024 Jan 23;9(4):919–28. doi: 10.1016/j.ekir.2024.01.035 (PMC11101778; doi:10.1016/j.ekir.2024.01.035)
Supplement: Supplementary File (PDF) [file mmc1.pdf]

Case	mode of delivery	PPH	pregnancy	preeclampsia	LDH (max)	Platelets (min)	Hb (min)	Acute dialysis	PEX	Renal pathology	Genetic analysis for susceptibility (pathogenic) variants for aHUS		CIT	time of FU	eGFR at last FU
no.		yes/no	weeks, fetal outcome	yes/no	U/l	×10³/µL	g/dl	yes/no	number		neg./pos.*	Genetic variant/ risk haplotype detected	weeks	months	ml/min (CKD-EPI)
**no PPH group**															
1	caesarian section	no	37, fetal death	no	1211	22	5,5	yes	yes, 3x	N/A	neg.	*CFH-H3: het.*	2	14	127
2	caesarian section	no	32	yes	4822	37	6,8	yes	no	TMA with ATN and ischemia	N/A		no	7	68
3	spontaneous delivery	no	N/A	yes	4297	27	5,1	no	no	N/A	neg.	none detected	no	0	120
4	spontaneous delivery	no	<20, missed abortion	no	1645	41	5,5	yes	yes, 6x	N/A	**pos.**	CFH: p.Glu1172* het	ongoing	57	47
5	caesarian section	no	33	yes	1131	63	12,1	no	no	N/A	neg.	risk-related haplotypes not reported	no	0	62
6	caesarian section	no	36	no	1460	55	5,1	yes	yes, 3x	acute TMA, severe ATN	neg.	risk-related haplotypes not reported	66	16	66
7	caesarian section	no	12	no	560	45	6,2	no	no	TMA with IgA-nephropathy.	neg.	*MCP-H2: hom.*	32	7	ESKD
8	caesarian section	no	33	no	1416	35	5,8	yes	yes, 22x	severe chronic and acute TMA	**pos.**	CFH: c.3493+5G>A, splice variant, Thrombomodulin: c.127G>A; *MCP-H2: hom.*	16	69	31
9	spontaneous delivery	no	25, fetal death	no	N/A	N/A	N/A	no	no	TMA	neg.	CFHR1 and CFHR3 deletion: hom.	no	5	59
10	spontaneous delivery	no	37	no	518	163	6,3	no	yes, 3x	TMA, severe ATN	neg.	*MCP-H2: het.*	25	6	73
11	spontaneous delivery	no	16, fetal death	no	576	32	6,3	yes	yes, 6x	severe TMA. Also catastrophic antiphospholipid syndrom (known Lupus erythematodes)	N/A		ongoing	2	ESKD
12	caesarian section	no	30	no	N/A	N/A	N/A	yes	no	severe and acute TMA, severe ATN	neg.	none detected	43	45	ESKD
13	caesarian section	no	39	no	2467	28	8,5	no	yes, 5x	N/A	**pos.**	CFH: c.58G>A p.; *MCP-H2: hom.*	ongoing	7	86
14	caesarian section	no	>30	yes	2773	38	6,2	yes	yes, 9x	N/A	neg.	none detected	no	7	>60
15	caesarian section	no	N/A	yes	N/A	N/A	N/A	no	yes, 2x	N/A	neg.	C3-VUS at c.1762G>A p(Asp588Asn); *MCP-H2: het.*	1	1	76

PPH group

16	caesarian section	yes	35	yes	>3000	43	N/A	yes	no	acute TMA, subtotal necrotic kidney tissue (seen as diffuse RCN)	neg.	CFHR3/CFHR1-Deletion: het;<br>*CD46-H2: het.;*<br>*CFHR1*B: hom.*	30	10	22
17	spontaneous delivery	yes	36	no	2686	47	5,7	yes	yes, 6x	N/A	neg.	CFHR3/CFHR1 deletion: het.;<br>*MCP-H2: het.*	147	37	27
18	caesarian section	yes	N/A	yes	2944	29	5,7	yes	no	N/A	neg.	CFHR3/CFHR1 deletion: het.;<br>*CHF-H3: het.;*<br>*CFHR1*B: hom.*	15	3	57
19	Abort curretage	yes	<20, missed abortion	no	2410	32	6,1	no	yes, 3x	N/A	neg.	*CFH-H3: het.*	39	19	104
20	caesarian section	yes	37	no	1809	57	6,5	yes	no	N/A	neg.	none detected	no	0	15
21	caesarian section	yes	36	no	3519	71	4,1	yes	yes, 2x	N/A	neg.	risk-related haplotypes not reported	74	28	85
22	caesarian section	yes	40	no	4639	20	6,2	yes	yes, 3x	subacute TMA, 40% diffuse RCN, severe ATN	neg.	risk-related haplotypes not reported	18	11	20
23	caesarian section	yes	N/A	no	2332	39	5,6	yes	yes,12x	N/A	neg.	risk-related haplotypes not reported	ongoing	2	74
24	caesarian section	yes	37	no	1407	26	5,9	no	no	acute TMA, severe ATN	neg.	risk-related haplotypes not reported	no	11	74
25	caesarian section	yes	16, fetal death	no	1209	66	5,3	no	yes, 4x	N/A	neg.	risk-related haplotypes not reported	1	15	115
26	caesarian section	yes	38	no	2402	59	6,3	no	no	N/A	neg.	risk-related haplotypes not reported	no	17	81
27	caesarian section	yes	32	yes	522	52	7,4	no	yes, 2x	N/A	neg.	risk-related haplotypes not reported	12	16	130
28	spontaneous delivery	yes	42	no	1347	35	5,6	yes	yes, 3x	N/A	neg.	risk-related haplotypes not reported	16	5	98
29	caesarian section	yes	41	no	1931	39	4,9	no	yes, 4x	TMA with ATN	neg.	*MCP-H2: het.*	no	1	78
30	spontaneous delivery	yes	40	no	3733	24	6	no	yes, 5x	N/A	neg.	none detected	11	24	93
31	spontaneous delivery	yes	N/A	no	796	163	5,9	yes	yes, 4x	N/A	neg.	none detected	63	17	63
32	caesarian section	yes	N/A	yes	2839	29	5,8	yes	yes, 4x	TMA with patchy cortical necrosis	neg.	*MCP-H2: het.*	223	52	33
33	caesarian section	yes	38	no	372	N/A	3,9	yes	yes, 6x	N/A	neg.	none detected	ongoing	23	80
34	caesarian section	yes	N/A, fetal death	no	947	18	5,1	yes	yes, 4x	severe ATN, no TMA	neg.	none detected	29	1	82
35	caesarian section	yes	35	no	513	56	7,1	yes	yes, n/a	TMA	neg.	*MCP-H2: hom.*	26	22	81

36	caesarian section	yes	32, fetal death	yes	4460	58	6	yes	yes, n/a	Acute TMA and patchy RCN	neg.	*MCP-H2: het.*	no	2	ESKD
37	caesarian section	yes	39, fetal death	yes	645	32	5,5	no	no	N/A	neg.	*MCP-H2: het.*	no	19	68
38	caesarian section	yes	37	yes	542	N/A	4,5	yes	yes, 5x	no TMA	neg.	CFHR1 and CFHR3 deletion: hom.	1	19	ESKD
39	Caesarian section	yes	37	no	3555	39	3,5	yes	Yes, 2x	TMA, diffuse RCN with 80% of tubuli with infarction	neg.	none detected	15	2	30
40	spontaneous delivery	yes	41	no	1944	21	7,4	no	Yes, 1x	No TMA but signs of hemolysis-induced tubulopathy	neg.	none detected	5	1	72

Supplement Table 1. Individual patient characteristics.

Individual courses and outcomes of patients presenting RCN on biopsy:

Case no. 16: The histological picture of subtotal necrotic kidney tissue was interpreted as diffuse RCN. This patient received no plasma exchange (PEX) but 7 months of CIT and recovered kidney function with an eGFR of 22 ml/min/1.73 m2 after 10 months.

Case no. 22: The biopsy showed proven patchy RCN and subacute TMA. The patient received three sessions of PEX and CIT for 4 months and recovered kidney function to an eGFR 20 ml/min/1.73 m² (Supplemental Table 1).

Case no. 32: The biopsy showed patchy cortical necrosis and the patient received four PEX treatments before receiving CIT for approximately 4 years. This patient recovered kidney function to an eGFR of 33 ml/min/1.73 m2.

Case no. 36: The biopsy showed patchy renal cortical necrosis and TMA; CIT was administered for unspecified reasons. The patient did not recover renal function.

Case no. 39: The biopsy showed 80 % renal cortical necrosis. The patient underwent two PEX sessions and continued treatment with CIT for 15 weeks. KRT was halted after 9 months because she experienced a partial renal recovery to an eGFR of 30 ml/min/1.73 m².

*neg./pos.: Indicates if a susceptibility (pathogenic) genetic variant for aHUS has been detected. N/A indicates that no analysis has been done.

PEX, plasma exchange. CIT, complement inhibitor treatment. eGFR, estimated glomerular filtration rate. LDH, lactate dehydrogenase. FU, follow-up. ESKD, end-stage kidney disease. ATN acute tubular necrosis. TMA thrombotic microangiopathy.

Patient variables	Total	no CIT	with CIT	p-value
Number of patients, **all patients**, n (%)	40 (100.0)	11 (27.5)	29 (72.5)	
Need for acute KRT, n (%)	24/40 (60.0)	4/11 (36.3)	20/29 (68.9)	0.08
GFR at end of follow-up (ml/min*1.73m²; CKD-EPI), mean ± SD	61.4 ± 36.4	62.3 ± 32.0	61.1 ± 38.5	0.93
Duration of KRT (weeks), median (IQR)	3.9 (1.3-20.0)	1.6 (0.7-3.0)	4.1 (1.4-21.5)	0.17
KRT at end of FU, n (%)	5/24 (20.8)	1/4 (25.0)	4/20 (20.0)	1.00
Receiving PEX treatment, n (%)	30/40 (75.0)	5/11 (45.5)	25/29 (86.2)	**0.014**

Supplement Table 2. Patient characteristics in women with and without complement inhibitor treatment (CIT). KRT, kidney replacement therapy. PEX, plasma exchange. eGFR, estimated glomerular filtration rate. SD, standard deviation.

Patient variables	Total	no CIT	with CIT	p-value
Number of patients**, no PPH**, n (%)	15 (100.0)	5 (33.3)	10 (66.6)	
Need for acute KRT, n (%)	8/15 (53.3)	2/5 (40.0)	6/10 (60.0)	0.60
KRT at end of FU, n (%)	3/8 (37.5)	0/2 (0.0)	3/6 (50.0)	0.46
Receiving PEX treatment, n (%)	10/15 (66.6)	2/5 (40.0)	8/10 (80.0)	0.25

Supplement Table 3. Patient characteristics in women in the no PPH group, with and without complement inhibitor treatment (CIT). KRT, kidney replacement therapy. PEX, plasma exchange. eGFR, estimated glomerular filtration rate. SD, standard deviation. FU follow-up.

Complement factors	Total	low	normal	high
**Activity classical pathway**	15	5	8	2
**Activity alternative pathway**	18	3	9	6
**C3d protein**	17	12	1	4
**sC5b-9 complex**	16	0	5	11
**C3 protein**	23	12	11	0
**C4 protein**	23	5	17	1

Supplement Table 4: Complement laboratory. Complement activity. Activity classical pathway (norm: 74-151%); Activity alternative pathway (norm: 60-140%); C3d <40 mU/L; sC5b-9 58-239; C3 0,89 - 1,87; C4 0.165 - 0.38
